# Immunocompatibility in transplantation: adapting to a changing therapeutic landscape

**DOI:** 10.3389/fimmu.2025.1648534

**Published:** 2025-09-09

**Authors:** Raquel Güell-Alonso, Raquel Cabrera-Pérez, Joaquim Vives

**Affiliations:** ^1^ Banc de Sang i Teixits, Barcelona, Spain; ^2^ Vall d’Hebron Research Institute (VHIR), Universitat Autònoma de Barcelona (UAB), Barcelona, Spain; ^3^ Department of Medicine, Universitat Autònoma de Barcelona (UAB), Barcelona, Spain

**Keywords:** ABO, emerging therapies, HLA match, immunosupression, personalized medicine, rejection, tolerance, transplantation

## Abstract

The ongoing shortage of cell, tissue, and organ donors has led to prioritizing clinical urgency over optimal immune matching in transplantation, often at the cost of increased reliance on immunosuppressive regimens and their associated adverse effects. Recent advances in the use of Substances of Human Origin (SoHOs), xenotransplantation and emerging cell-, gene-, and tissue-based therapies have enabled the development of tailored cellular therapeutics to enhance engraftment, long-term function, and immunological compatibility. Within this evolving context, artificial intelligence is also increasingly contributing to improve donor–recipient matching through predictive analytics and integrative data modeling, assisting on immune tolerance and the durable integration of transplanted cells into host tissues. In this review, we revisit foundational concepts of immunocompatibility, examine current clinical criteria in organ transplantation, and critically explore the shifting paradigms of donor–recipient matching in the era of personalized medicine. These advances have the potential to redefine clinical strategies in transplantation and regenerative care while ensuring patient access and sustainability.

## Graphical Abstract

Evolving paradigms in allogeneic transplantation. Part of the figure was designed using images from the NIH BIOART source (https://bioart.niaid.nih.gov/).

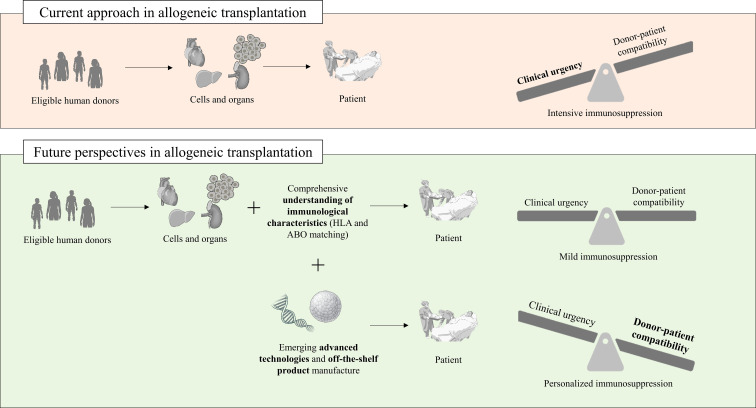

## 1 Introduction

Nowadays, the availability of donated cells, tissues, and organs is still insufficient to meet clinical needs, despite the enormous efforts made at many levels (medical, ethical, regulatory, logistical, health systems, and donation). Such persistent organ, stem cell, and tissue shortages necessitate innovative strategies aimed at the expansion of the donor pool by exploring alternatives while ensuring patient safety. Post-transplant complications such as graft-versus-host disease (GvHD) in hematopoietic stem cell transplantation (HSCT), chronic rejection, donor-specific alloantibody (DSA) formation, and sensitization continue to challenge the field, underscoring the need for ongoing investigation into the critical impact of compatibility issues between donor and recipient. This challenge can be addressed by a combination of emerging therapies and computational tools involving a deeper understanding of the thresholds at which donor-patient disparity can be deemed acceptable, with improved immunosuppressive approaches, thus offering new possibilities for increasing transplantation rates and advancing the frontiers of personalized medicine (1–4).

Herein we aim to introduce the major actors in donor-patient compatibility, understand their role in graft survival and tolerance, and discuss promising strategies aimed at a more accessible, equitable, customized, and sustainable transplantation framework. For clarity, **Table 1** includes definitions of terminology used in this article.

**Table 1:** Definitions.

CONCEPT	DEFINITION
*Antibody-mediated rejection (AMR)*	A form of graft rejection driven by the patient’s antibodies, most commonly donor-specific anti-HLA antibodies (DSAs), which target antigens on the vascular endothelium of the transplanted organ. AMR leads to complement activation, inflammation, and vascular injury, and is the major cause of graft dysfunction and loss, especially in kidney and heart transplants. Treatment requires intensive immunosuppression, plasmapheresis, and/or B-cell-targeted therapies.
*Donor-specific antibodies (DSAs)*	DSAs are recipient-derived antibodies that specifically recognize and bind to both HLA and non-HLA antigens expressed by the donor graft. They may be present before transplant (pre-formed as a result of sensitization from prior transplants, transfusions or pregnancy) or may develop *de novo*. DSAs are a key driver of AMR and are associated with poor transplant outcomes, including allograft rejection, dysfunction, and loss. Monitoring DSAs is essential for risk stratification, graft surveillance, and guiding immunosuppressive therapy.
*Eplet analysis*	Eplet analysis is a high-resolution immunogenetic method for assessing mismatches between donor and recipient HLA alleles at the epitope level. Unlike traditional allele-level matching, eplet analysis focuses on structural amino acid configurations on the surface of HLA molecules that are recognized by B-cell receptors and antibodies. This approach allows the assessment of immunologic risk, predicting DSA formation and guiding precision immunosuppression and donor selection strategies. Eplet mismatching is increasingly being used in both solid organ and HSCT to improve long-term graft outcomes.
*Graft-versus-host disease (GvHD)*	GvHD is a serious complication that can occur after allogeneic hematopoietic stem cell transplantation (HSCT), in which donor immune cells (especially T cells) recognize the recipient’s tissues as foreign and mount an immune response against them. This immune attack can target organs such as the skin, liver, and gastrointestinal tract. GvHD is classified as acute or chronic based on the timing of the onset and clinical features. Although harmful, mild GvHD can also reflect a beneficial graft-versus-leukemia (GvL) effect, helping prevent cancer relapse.
*Haploidentical transplant*	A type of allogeneic HSCT with a half-matched (haploidentical) related donor, typically sharing 50% of the HLA alleles with the recipient. This approach broadens the pool of potential donors when a fully matched donor is not available and has become increasingly feasible due to advancements in graft manipulation and post-transplant immunosuppressive strategies (such as post-transplant cyclophosphamide, PTCy) to reduce GvHD and rejection.
*Immunosuppressant*	Agent that reduces or inhibits the activity of the immune system, primarily used to prevent or treat rejection in organ and tissue transplantation, or to manage autoimmune diseases. These agents reduce the immune response to alloantigens or self-antigens but often increase susceptibility to infections and malignancies as a consequence of generalized immune suppression.
*Sensitization*	Immunological process whereby an individual develops alloantibodies, particularly anti-HLA antibodies, following exposure to foreign antigens through events such as blood transfusions, pregnancy, or previous transplants. This immune priming increases the risk of graft rejection and complicates future transplantation by narrowing the pool of compatible donors.
*Virtual cross-matching*	Virtual cross-matching is a pre-transplant immunological assessment that predicts the compatibility between a donor and recipient without physically mixing their blood samples. It is becoming a cornerstone in modern transplant immunology, particularly in kidney and heart transplantation. This approach relies on detailed HLA typing of both the donor and recipient, along with the recipient’s known HLA antibody profile (typically identified through Luminex-based assays) to determine whether the recipient has pre-formed antibodies against the donor’s HLA antigens, which could lead to hyperacute or acute AMR. A negative virtual crossmatch suggests a low immunological risk and may expedite organ allocation, particularly in urgent or geographically distant situations.

## 2 Actors in graft rejection

The immunological rejection of transplanted cells, tissues, and organs is orchestrated by a complex interplay of cellular and humoral immune responses, primarily governed by recognition of non-self antigens.

**Figure 1:**
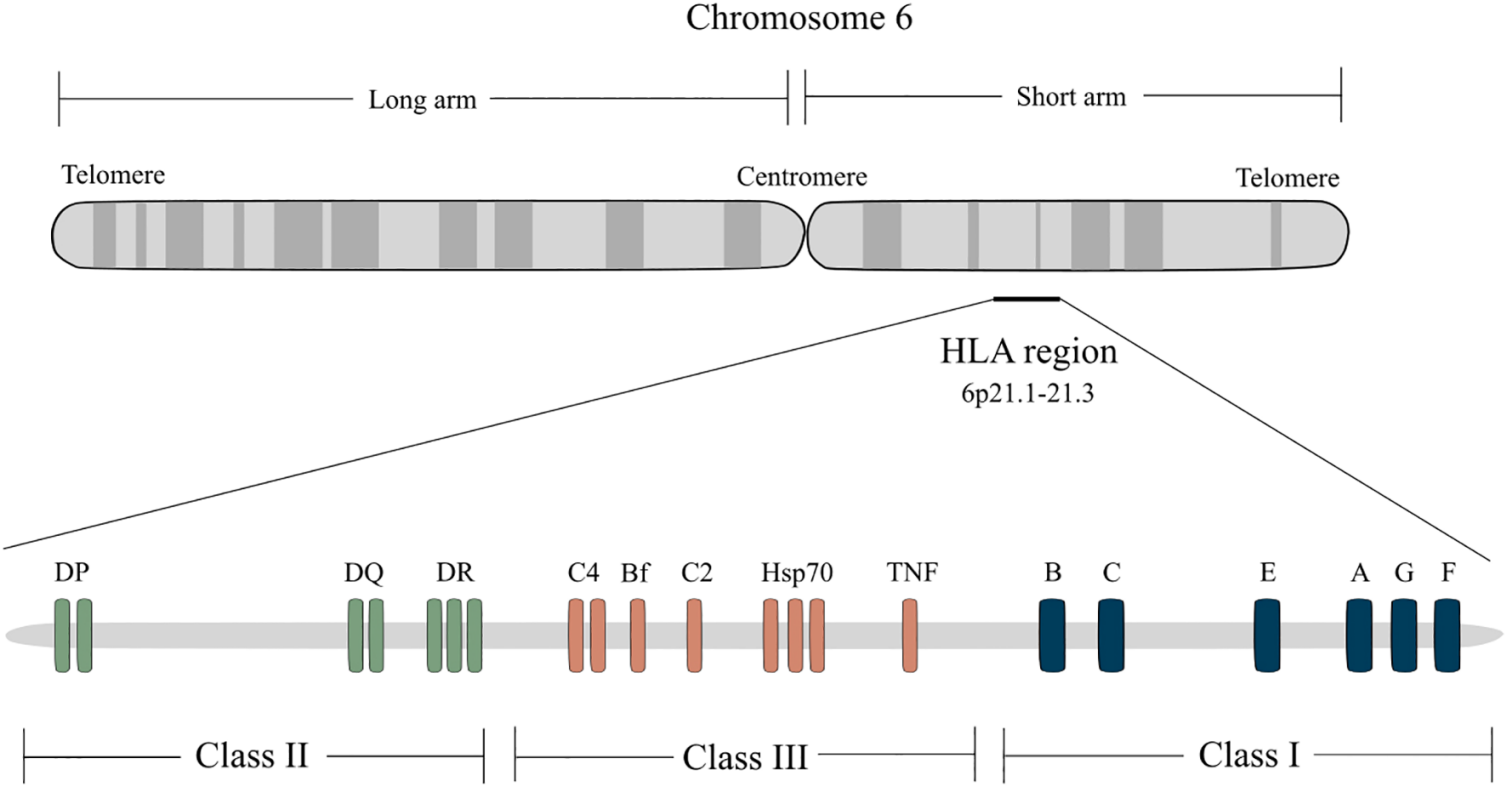
Classical and non-classical HLA loci. The human major histocompatibility complex (MHC) is called the HLA (human leukocyte antigen) and is located on the short arm of chromosome 6 (6p21.1-21.3). The class I region contains the classical *HLA-A*, *HLA-B*, and *HLA-C* genes that encode the heavy chains of class I molecules, which present antigens to CD8^+^effector T cells, and the non-classical *HLA-E, HLA-F*, and *HLA-G*, which interact with NK cells. The class II region consists of a series of subregions, namely *DR*, *DQ*, and *DP*, each containing *A* and *B* genes encoding α and β chains, respectively. Class II reactive T cells are usually CD4^+^ helper cells. HLA class I molecules are expressed on the surface of almost all nucleated cells while class II molecules are expressed only on B lymphocytes, APCs (monocytes, macrophages, and dendritic cells), and activated T lymphocytes. The class III region does not encode HLA molecules but other important genes, including C’ (complement genes), HSP (heat shock protein) and TNF (tumor necrosis factor).

The major histocompatibility complex (MHC) system, known as the human leukocyte antigen (HLA) in humans, is central to this process and represents the most polymorphic region of the human genome (**Figure 1**). This high degree of polymorphism makes each individual almost immunogenetically unique, critically determining graft tolerance (5). Classical MHC class I (HLA-A, -B, -C) and class II (HLA-DP, -DQ, -DR) molecules both present alloantigens to recipient T lymphocytes, initiating direct or indirect allorecognition pathways that trigger effector immune responses and contribute to chronic rejection, reduced graft survival, and severe complications. Beyond the classical HLA loci, increasing evidence highlights the role of non-classical MHC-I molecules such as *i)* HLA-G, which exerts immunomodulatory effects and is implicated in maternal-fetal tolerance; and *ii)* HLA-E, which interacts with innate and adaptive immune cells leading to both protective and detrimental effects on allograft survival (6–9). Similarly, minor histocompatibility antigens (mHAgs), although less immunogenic than HLA molecules, can also trigger GvHD after HSCT and contribute to late rejection events.

Traditional HLA matching has largely focused on major immunogenic HLA loci, namely HLA-A, -B, and -DR. Matching at the HLA-DR locus has been shown to have the most pronounced effect on allograft survival and long-term graft function (10). However, mismatches in HLA-DP and -DQ are also relevant, especially in highly sensitized patients or those undergoing multiple transplants.

The ABO blood group also represents another key immunological barrier, particularly in solid organ transplantation, where naturally occurring antibodies against non-self ABO antigens can mediate hyperacute rejection and graft dysfunction in incompatible settings. The *ABO* gene encodes for a glycosyltransferase that modifies oligosaccharides on the surface of the red blood cells (RBC), vascular endothelium, and other tissues (11). Variations in the sequence of *ABO* are responsible for the major blood group phenotypes (A, B, AB, and O). Interestingly, more than 300 RBC antigens belonging to 36 blood group systems have been officially reported in humans by the International Society of Blood Transfusion (ISBT) so far (12). ABO-incompatible transplantation is feasible but requires desensitization and intensification of immunosuppression to prevent allograft rejection (11).

Additionally, a number of immune cell types participate in graft acceptance or failure. Antigen-presenting cells (APCs), including dendritic cells, macrophages, and B cells, play an initiating role by processing and presenting alloantigens to naïve T cells, thus bridging innate and adaptive immunity. Effector CD8^+^ cytotoxic T cells and CD4^+^ helper T cells, along with natural killer (NK) cells, plasma cells, and memory B cells, perpetuate graft rejection via direct cytotoxicity, cytokine release, and alloantibody production (13).

Finally, the interplay between these actors is further shaped by the inflammatory *milieu*, the immune status of the recipient (e.g., pre-sensitization), and the immunogenicity of the graft itself, which are all important factors in innovative cell-based therapies, tissue-engineering, and xenogeneic applications. As transplantation strategies evolve toward precision immunomodulation, a better understanding of these actors at the cellular, molecular, and epitope-specific levels becomes imperative to predict and prevent rejection, tailor immunosuppression and, ultimately, enhance long-term graft survival.

## 3 Compatibility in transplantation

**Figure 2:**
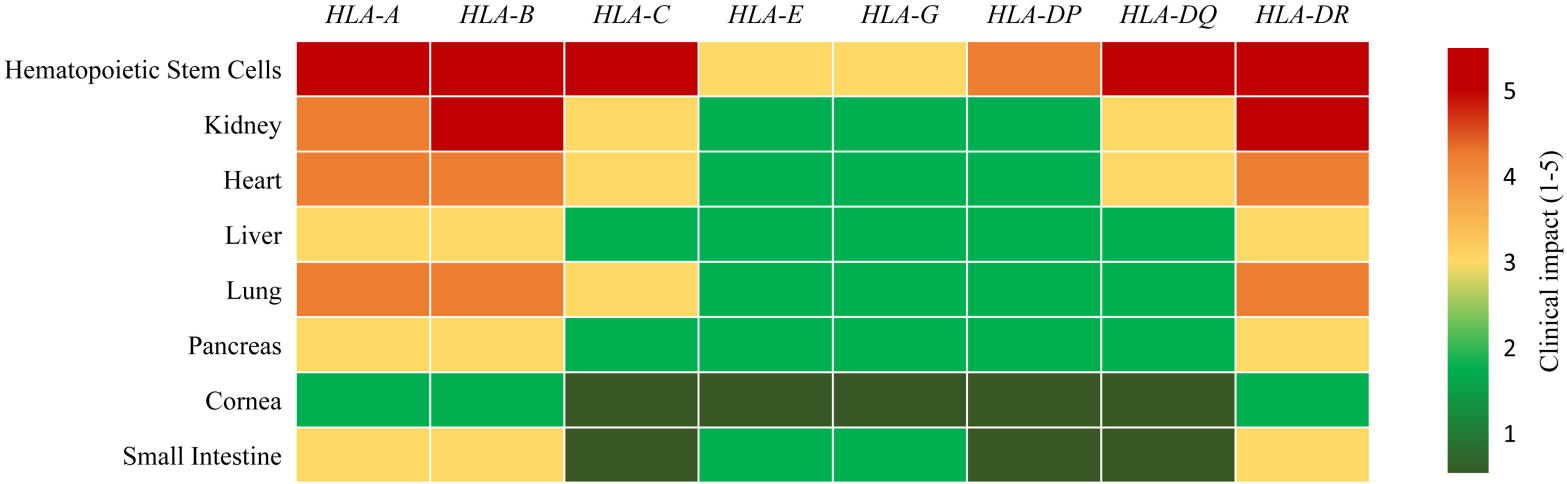
Relevance of donor-recipient HLA loci compatibility in organ transplant success. A higher score (5, shown in red) indicates that matching at the given HLA locus is critically important for transplant success, whereas a lower score (1, shown in dark green) indicates lower clinical impact in current transplant settings in combination with immunosuppressive agents.

Stringent HLA matching is often balanced against the urgency of transplantation and donor availability. The entire lifespan of an organ transplant recipient relies on efforts to maintain the delicate balance between the risk of rejection on the one hand, and on the other, the risk of infection and malignancy later on. Although the current intensive immunosuppressive protocols reduce the occurrence of severe acute rejections (ARs) to a minimum, patients with functioning organs may later die of severe infections or malignancies instead. In the following sections, we present current evidence on the minimal HLA donor-recipient match requirements as well as the influence of ABO blood types for successful outcomes across major organs and tissues (summarized in **Table 2** and **Figure 2**).

**Table 2:** Current criteria on HLA and ABO compatibility considerations in major organ transplantation. .

SoHO	Compatibility		Immunosuppression	Complications	Comments
	HLA	ABO			
*HSC*	+++	+	++	Rejection, GvHD	Registries allow for the rapid and efficient search for highly compatible donors. Haploidentical transplants and PTCy expand donor availability.
*Kidney*	++	++	++	AMR	Crossmatching and DSA testing are crucial. Acceptable mismatches depend on sensitization and donor availability. HLA matching is prioritized in living donor kidney transplantation or younger recipients.
*Heart*	–	++	+++	Rejection, immunosuppression-related complications	HLA matching can improve outcomes by reducing sensitization and chronic rejection, but logistical challenges and the urgency of these transplants often outweigh strict HLA compatibility. Intensive use of immunosuppressants.
*Liver*	–	++	+	Rejection	The liver is immunologically privileged, so HLA matching plays a minimal role in most cases. ABO compatibility is far more critical.
*Lung*	–	–	++	Rejection, CLAD, BOS	HLA and ABO matching can improve outcomes. However, perfect matching is not strictly required due to issues with organ availability.
*Pancreas*	+	++	++	Rejection	Success is more reliant on careful immunosuppression and ABO compatibility. Pancreatic islet cell transplants often require less stringent matching.
*Cornea*	–	–	–		Due to the immune-privileged status of the corneal tissue, corneal transplants are not usually influenced by HLA matching, except in high-risk cases (vascularized tissue, previous graft rejection, or active inflammation). In such high-risk patients, ABO compatibility is also prioritized.
*Small Intestine*	–	++	+++	Rejection, GvHD, immunosuppression-related complications	HLA matching is ideal to improve graft survival and reduce rejection risks. However, due to the scarcity of donors, practical application of HLA matching is limited, and immunosuppressive protocols play a pivotal role.

AMR (antibody-mediated rejection); BOS (bronchiolitis obliterans syndrome); CLAD (chronic lung allograft dysfunction); DSA (donor-specific antibodies); GvHD (graft-versus-host disease); PTCy (post-transplant cyclophosphamide); SoHO (substances of human origin). Level of relevance: insignificant (-), low (+), medium (++), and high (+++).

### 3.1 Hematopoietic stem cells

HSCT is indicated for hematologic malignancies, non-malignant blood disorders, primary immunodeficiencies, severe autoimmune diseases, and congenital metabolic disorders, serving as a curative or life-extending therapy by replacing dysfunctional hematopoietic and immune systems (14). In HSCT, HLA matching (especially at HLA-A, -B, -C, and -DR) is paramount to prevent graft failure, GvHD, and other complications. A 10/10 match (for both alleles of HLA-A, -B, -C, -DR, and -DQ) is preferred for unrelated donor transplants, while mismatches at a single locus (e.g., 9/10) are acceptable but increase the risk of GvHD. It is worth noting that having national and international registries enables highly compatible donors to be identified rapidly and efficiently. Patients lacking HLA-identical donors can be treated with haploidentical transplants that allow the use of HSC from an allogeneic half-matched donor (5/10 match), who is typically a family member. However, they often require additional interventions to mitigate risks, such as T-cell depletion or post-transplant immunomodulation. HLA-C and HLA-DP matching has also been shown to improve outcomes in HSCT (15).

### 3.2 Kidney

Kidney transplantation is indicated for patients with end-stage renal disease (ESRD) or advanced chronic kidney disease (CKD) who present irreversible loss of kidney function, requiring renal replacement therapy to improve survival and quality of life. Cross-matching and DSA testing are crucial adjuncts to HLA matching for kidney transplants. Acceptable mismatches depend on sensitization and availability of donors. HLA-A, -B and -DR matching has a direct impact on long-term graft survival, while HLA-DR mismatches tend to be the most immunogenic and impactful. HLA-B mismatches also carry a strong risk due to high polymorphism. HLA-A mismatches are important but generally considered less immunogenic than -DR or -B. Patients with higher HLA matches require less immunosuppression and have a reduced risk of AMR. HLA matching is often prioritized in living donor kidney transplantation or for younger recipients where longevity of the graft is critical (16).

Interestingly, ABO antigens are expressed on kidney endothelial and epithelial surfaces, and their presence on allograft tissue can lead to higher short-term hyperacute or acute AMR, although with good management, long-term outcomes can approach those of compatible transplants. It is worth noting that blood group B kidney recipients experience longer waiting times than other ABO groups prior to transplantation (17). The prevalence of group B is higher in African Americans and Asian Americans and thus these ethnic minorities are the most affected populations within the group B cohort (18).

Apart from HLA/ABO matching, the effect of other factors on post-transplant outcomes, including viral serology, age and size mismatch, mismatch in other HLA antigens (e.g. HLA-C and HLA-DQ), and eplet matching, has become increasingly recognized. Of note, kidney transplant recipients who experience rejection can return to dialysis, a situation that is not paralleled in other transplantation settings.

### 3.3 Heart

Heart transplantation is indicated for patients with end-stage heart failure due to conditions such as dilated or ischemic cardiomyopathy, congenital heart disease, or refractory ventricular arrhythmias, who have severe symptoms despite medical, surgical, or device-based therapies and meet criteria for irreversible hemodynamic compromise. Although, where feasible, HLA-A, -B, and -DR matching is considered, primarily to reduce the risk of sensitization in re-transplantation cases, no specific HLA match requirement is universally mandatory given that transplantation may not be delayed due to clinical urgency. Consequently, immunosuppression protocols are heavily relied upon in heart transplants, much more than in kidney transplantation, since graft rejection is usually fatal (19). This may explain why no further decline in the organ survival rate was found as the number of HLA mismatches increased from three to six (19, 20). As a consequence of intensive immunosuppressive treatment, high rates of malignant lymphomas are reported in heart transplant patients (21).

Transplantation of hearts from ABO-incompatible donors is contraindicated because of the risk of hyperacute rejection. This contraindication may not apply to infants younger than 2 years, who do not yet produce antibodies against T-cell-independent antigens. However, use of donors with minor ABO mismatches is also a safe and feasible option in older children and adult patients (22–24).

### 3.4 Liver

Liver transplantation is indicated for patients with end-stage liver disease, acute liver failure, or metabolic disorders affecting hepatic function due to conditions such as cirrhosis, hepatocellular carcinoma within transplant criteria, or genetic liver diseases, when no alternative treatment can prevent life-threatening complications or hepatic decompensation. In liver transplantation, no stringent HLA matching is required since the liver is considered immunologically privileged compared to other organs due to its inherent tolerogenic properties. Of note, ABO compatibility is more critical than HLA matching for liver transplants (25–27).

### 3.5 Lung

Lung transplantation is indicated for patients with end-stage pulmonary disease, such as chronic obstructive pulmonary disease (COPD), idiopathic pulmonary fibrosis, cystic fibrosis, or pulmonary arterial hypertension, who have severe respiratory failure and reduced life expectancy. Matching at HLA-A, -B, and -DR may improve long-term outcomes, particularly chronic lung allograft dysfunction (CLAD) and bronchiolitis obliterans syndrome (BOS). However, issues related to organ availability mean that perfect matching is not strictly required. High levels of pre-transplant sensitization increase the risk of rejection. Opelz and collaborators analyzed a large series of lung transplant procedures followed up on for 5 years, demonstrating that a high number of HLA mismatches or, surprisingly, zero mismatches, unfavorably impacts graft survival rates (28). Recently, HLA-C mismatches have been described as beneficial in lung transplantation due to a reduced incidence of CLAD in recipients with HLA-C mismatching (29).

### 3.6 Pancreas and pancreatic islets

Pancreas and pancreatic islet transplantation are indicated for patients with type 1 diabetes mellitus experiencing severe glycemic instability, recurrent hypoglycemia unawareness syndrome, or secondary complications, particularly when simultaneous kidney transplantation is required due to ESRD or when intensive insulin therapy fails to achieve metabolic control. HLA-A, -B, and -DR matching is ideal in pancreas transplantation but is not always feasible. According to the results published by Rudolph et al., the risk of acute rejection increases significantly at four or more mismatches, especially when they involve the HLA-B or -DR locus (30). In contrast, recent results suggest that HLA matching is not associated with improved graft survival or a reduction in acute rejection (31).

Interestingly, success in pancreas transplantation is more reliant on careful immunosuppression and ABO compatibility. Despite this, there have been successful cases of ABO-incompatible pancreas transplantation, particularly in simultaneous pancreas-kidney (SPK) procedures. These cases usually involve intensive immunosuppressive regimens and pre-transplant antibody removal strategies (e.g., plasmapheresis, rituximab) to lower the risk of rejection.

Pancreatic islet cell transplants often require less stringent matching due to *i)* advances in immunosuppressive options, and *ii)* the infusion of a small amount of islets into the liver via the portal vein. In this context, a recent study suggests that recipients with HLA-DR matching, excluding diabetogenic HLA-DR3 and -DR4 alleles, maintained higher rates of insulin independence 5 years after transplantation compared to those with mismatching (32). Of note, the purity of islet preparations has a direct impact on rejection due to contamination with pancreatic exocrine tissue components and acinar tissue expressing ABH antigens (33). Additionally, certain HLA-DQ antigens have been associated with improved graft survival (34).

### 3.7 Cornea

Corneal transplantation, also known as keratoplasty, can be complete (penetrating keratoplasty) or partial (endothelial keratoplasty) and is indicated for patients with corneal opacification, thinning, or structural damage due to conditions such as keratoconus, corneal dystrophies, infections, trauma, or scarring, when visual rehabilitation or ocular integrity cannot be achieved through other interventions. Although no HLA matching is typically required for low-risk corneal transplants due to the immune-privileged status of the corneal tissue (characterized by avascularity, low expression of HLA antigens, and presence of immunomodulatory molecules), HLA-A, -B, and -DR matching can reduce the risk of rejection in high-risk cases (e.g., vascularized tissue, previous graft rejection, or active inflammation) (35–37). In such high-risk patients, ABO compatibility is also prioritized.

### 3.8 Small intestine

Small bowel transplantation, which is a relatively infrequent procedure, is indicated for patients with irreversible intestinal failure who are unable to maintain adequate nutrition and hydration through parenteral nutrition due to life-threatening complications such as liver failure, recurrent sepsis, or loss of central venous access. The small intestine is highly immunogenic and has one of the highest rates of acute rejection among all solid organ transplants (38). HLA-A, HLA-B, and, particularly, HLA-DR matching is ideal to improve graft survival and reduce the risk of rejection (39). However, due to the paucity of donors, practical application of HLA matching is limited, and immunosuppressive protocols play a pivotal role (38). These factors place patients at increased risk of developing immunosuppression-related complications, namely cellular rejection and AMR, infection, kidney disease, lymphoproliferative disorders, and GvHD.

ABO compatibility is usually mandatory, except for pediatric patients under 1 year old. Nevertheless, ABO-incompatible transplants could be feasible with the appropriate management of blood type antibodies and the use of adequate immunosuppression in the early period (40).

## 4 Impact of emerging technologies

The notion of immunocompatibility in transplantation is being redefined by a convergence of biomedical innovations, such as advances in molecular diagnostics, gene editing, and regenerative medicine, which go beyond traditional HLA and ABO matching paradigms. These technologies have begun to reshape not only how compatibility is assessed but also how immune risk is managed and donor availability is expanded. Broadly, three strategic frameworks have emerged: *i)* tools to increase compatibility precision; *ii)* modalities to broaden donor resources; and *iii)* interventions to mitigate adverse immune reactions. While their application varies across transplant settings, these strategies reflect a shift toward dynamic, tailored approaches in clinical transplantation.

### 4.1 Enhancing compatibility precision

A first group of technologies focuses on refining histocompatibility assessment and improving donor–recipient matching at molecular and functional levels. On the one hand, RNA-based next-generation sequencing (NGS) and advanced bioinformatics pipelines now enable allele-specific HLA expression profiling, providing deeper insight into immune compatibility, informing donor selection more accurately, and improving predictive modelling for transplant outcomes. These techniques are especially relevant in HSCT (14).Similarly, high-resolution typing and eplet analysis have advanced compatibility beyond antigen-level matching. Algorithms such as *HLA-Matchmaker*, *PIRCHE*, and *HLA-EMMA* analyze amino acid polymorphisms and antigen-presenting peptide predictions to quantify mismatches more precisely, enabling clinicians to stratify risk at the molecular interface of HLA. These tools are particularly valuable in heart transplantation, where nuanced HLA compatibility influences graft survival and immunosuppression needs (18).

Additionally, artificial intelligence (AI) and machine learning (ML) have revolutionized donor matching and risk stratification. In HSCT, predictive models integrate HLA typing with clinical parameters such as patient comorbidities, disease stage, and immunogenetic factors to identify optimal donors (41). In kidney transplantation, AI and ML tools -including Chatbot- assist throughout the transplant process, from donor selection to postoperative monitoring (42). Other quantitative tools such as the *Living Kidney Donor Profile Index (LKDPI)* empirically compares potential living donors across multiple factors (viral serology, age, eplet matching) to more precisely characterize donor-recipient incompatibilities and improve long-term graft survival (43). In liver transplantation, ML models can be used to identify patients at high risk for developing GvHD and to predict graft failure (44–46). For lung transplantation, *InsightTx* employs XGBoost algorithms to predict outcomes based on *ex vivo* lung perfusion (EVLP) data (46, 47). Similarly, pancreas transplantation has benefited from Naive Bayesian Classifier and Support Vector Machine-based models to estimate rejection probability in simultaneous pancreas–kidney recipients (48). In corneal transplants, AI applications utilizing Optical Coherence Tomography (OCT) imaging have also demonstrated remarkable accuracy in evaluating graft rejection (49).

Finally, gene editing technologies also serve compatibility goals by directly modifying donor organs or cells to enhance compatibility at the genetic level. CRISPR/Cas9 tools allow targeted disruption of immunogenic loci (e.g., HLA, T cell receptor TCRα), insertion of safety or regulatory transgenes (e.g., suicide switches, cytokine modulators), and overexpression of immunotolerance-associated molecules (e.g., HLA-E, CD47). These interventions are being explored in heart and lung transplantation. In this field, Figueiredo and collaborators proposed silencing donor MHC molecules via shRNAs to reduce immunogenicity, while CRISPR/Cas technologies hold promise for generating universal blood type lungs (50–52).

### 4.2 Expanding the donor pool

While compatibility precision improves outcomes, donor scarcity remains a fundamental constraint, especially for time-sensitive organs. To address this, a spectrum of strategies has emerged to expand transplantable resources without compromising immunological feasibility.

In this context, xenotransplantation has made significant strides, particularly with porcine donors genetically modified to silence xenoantigens involved in hyperacute rejection (synthesizing α-1,3-galactosyltransferase, *GGTA1*; and β-1,4-N-acetyl-galactosaminyltransferase 2, *B4GALNT2*), and synthesizing N-glycolylneuraminic acid (*CMAH*)) and express human transgenic proteins that regulate immune response (CD47 and heme oxygenase 1), coagulation (human thrombomodulin, hTBM), and complement activation (CD39, CD46 and CD55). Proof-of-concept transplants of porcine kidney, heart, and liver into human recipients have shown early success, suggesting that compatibility can be manufactured, rather than matched (53–56).

Bioengineering and regenerative medicine approaches, such as tissue engineering and 3D bioprinting, are also expanding alternatives to traditional organ replacement while enabling the development of personalized therapies through the creation of functional organ substitutes. These strategies reduce dependence on donor availability and enable HLA-compatible solutions. Scaffolds from decellularized organs repopulated with human stem or allogeneic progenitor cells (such as in kidney and heart bioengineering) preserve the native extracellular matrix while removing immunogenic antigens, promoting regeneration and reducing immune rejection (57–60). Kidney and liver organoids mimicking native tissue architecture are also being developed to increase graft availability (61–63). Intestinal organoids derived from adult stem cells or iPSCs show promise for treating conditions like inflammatory bowel disease, celiac disease, and short bowel syndrome by restoring barrier integrity, modulating immune response, and supporting nutrient absorption. In mice, transplanted epithelial organoids integrate and promote mucosal healing, with some studies using hydrogels or decellularized scaffolds to engineer functional intestinal tissue (64–67). In pancreatic transplantation, alginate encapsulation of islets has been explored to reduce immune rejection, even without immunosuppression, in both allo- and xenotransplantation settings (68–72). In the kidney, 3D bioprinting technologies aim to recreate nephron-like structures for future renal replacement therapies (57, 73), while similar strategies in the liver and lung are used to fabricate transplantable hepatic tissue or airway structures, respectively (62, 74).

Lastly, *ex vivo* organ perfusion platforms such as normothermic machine perfusion (NMP) and EVLP extend the viability of marginal grafts and enable functional rejuvenation. These systems also act as delivery routes for immunomodulatory agents (e.g., MSCs) and gene vectors, thereby minimizing off-target effects and vector-induced inflammation and transforming preservation into a therapeutic window. Applied in kidney, heart, lung, and liver transplantation, perfusion technologies help bridge immunological gaps and optimize graft readiness (75–78).

### 4.3 Mitigating immune adverse effects

When HLA or ABO compatibility cannot be strictly achieved, technological advances aim to neutralize immunological consequences and promote tolerance. Innovation in immunosuppressive regimens includes post-transplant cyclophosphamide (PTCy), costimulatory blockade, monoclonal antibodies targeting memory B and T cells, and targeted drug delivery. In HSCT, PTCy has gained prominence as a cost-effective method with strong GvHD prevention capacity, significantly expanding the use of haploidentical donors and redefining donor selection criteria (79). In lung, upregulation of interleukin-10 (IL-10) in models of acute rejection and CLAD has been shown to reduce inflammation and improve graft tolerance (80, 81). In intestinal transplantation, therapies targeting key inflammatory mediators, such as monoclonal antibodies against TNF-α and integrins, proteasome inhibitors and purine analogs, and modulation of the intestinal microbiome through selective antibiotics or probiotics reduce rejection while minimizing systemic toxicity (82).

Besides pharmacological approaches, cell-based immunomodulation is nowadays at the forefront. Regulatory T cells (Tregs) are increasingly used in HSC, kidney, liver, pancreas, and small intestine transplantation to prevent rejection and promote immune adaptation (82–87). Moreover, mesenchymal stromal cells (MSCs) exhibit immunosuppressive and anti-inflammatory properties, with therapeutic use spanning HSC, liver, lung (where a first-in-human study showed a slower decline in lung function in patients with advanced CLAD after MSCs infusion), and small intestine transplants (84, 86, 88–91). Additionally, regulatory dendritic cells have also shown promise in liver immunoregulation (92) and, recently, PRDM16-dependent APCs have been described to induce tolerance to gut antigens, offering new insights into developing therapeutic strategies for intestinal transplant tolerance (93). Chimerism-based strategies have been also tested, promoting immune adaptation through the co-transplantation of donor HSCs, as demonstrated in kidney and liver transplantation (94, 95). Finally, CAR-engineered immune cells, including CAR-T and CAR-NK, are under investigation in HSCT for their dual role in eliminating residual disease and modulating post-transplant immunity (83, 84, 96). AI-assisted monitoring complements these interventions. Tools analyzing gene expression profiles (e.g., AlloMap), donor cfDNA levels, and longitudinal patient data help detect early signs of rejection and guide immunosuppression tapering, thus allowing real-time adaptation and adjusting drug regimens dynamically to avoid over- or under-immunosuppression (42, 97–102).

These converging innovations indicate a shift from rigid antigen matching toward a functional and personalized approach to immunocompatibility. By integrating molecular precision, regenerative capacity, and predictive analytics, emerging technologies offer viable pathways to reconcile immunological complexity with therapeutic feasibility in modern transplantation.

## 5 Final remarks and outlook

Transplantation medicine has entered a transformative phase where immunocompatibility is no longer governed by a one-size-fits-all framework. In HSCT and kidney transplantation, compatibility-enhancing platforms are already reshaping donor selection. These fields will likely remain at the forefront of high-resolution typing, algorithmic matching, and integration of gene-edited therapies. In contrast, heart and pancreas grafts, constrained by urgency and donor scarcity, may benefit more from immune engineering, scaffolded tissues, and xenogeneic sources. Although the liver is more immunotolerant, these innovations also offer complementary solutions where donor availability is limited. In lung and intestinal transplantation, technologies mitigating immune aggression (such as EVLP-based gene delivery, PRDM16-dependent tolerance, and MSC infusions) are addressing the limitations of conventional matching. Meanwhile, corneal transplantation and pancreatic islets, both involving immune-privileged or compartmentalized sites, exemplify how bioengineered constructs and smart encapsulation can circumvent systemic immune triggers.

Looking ahead, compatibility may become a design feature rather than a selection criterion. Organoids, bioprinted tissues, and modular graft systems could be engineered with specific immune profiles, built to engage host defenses intelligently or resist them entirely. AI-driven dashboards might one day manage graft–host communication proactively, guiding immunomodulation not by static protocols but by continuous biological feedback. In this landscape, HLA and ABO matching remain foundational, but no longer absolute. Compatibility is expanding beyond genetic coincidence to include dynamic tolerability, functional resilience, and engineered neutrality. The goal is not perfect alignment, but sustainable integration: building transplant systems that adapt, persist, and heal across the immunological spectrum.

By embracing this new paradigm, transplantation can move from the constraints of biological inheritance toward the possibilities of biomedical design, where the immune system is not an obstacle to be overcome, but a partner to be engaged through technology, insight, and innovation.
